# Reddit and rare diseases: what myositis communities tell us about support and struggle

**DOI:** 10.1093/oodh/oqag007

**Published:** 2026-04-11

**Authors:** Andrew S Wilson, Hamza Bin Sajid, Latika Gupta

**Affiliations:** Department of Computer Science, Birmingham City University, STEAMHouse, Belmont Row, Birmingham B4 7RQ, United Kingdom; Department of Computer Science, Birmingham City University, STEAMHouse, Belmont Row, Birmingham B4 7RQ, United Kingdom; Department of Rheumatology, Royal Wolverhampton Hospitals National Health Service Trust, Wolverhampton WV10 0QP, United Kingdom; School of Infection, Inflammation and Immunology, College of Medicine and Health, University of Birmingham B15 2TT, United Kingdom

**Keywords:** myositis, natural language processing, Reddit, sentiment analysis, topic modeling, digital health, patient support, rare disease

## Abstract

Myositis is a rare autoimmune condition associated with muscle weakness, systemic involvement, and long-term disability. People living with rare conditions frequently use online platforms to share experiences and seek information beyond formal healthcare settings. This exploratory study applied automated natural language processing (NLP) methods to analyse public posts and comments from the myositis subreddit between 2020 and 2024. The dataset comprised 1223 unique posts and 18 453 unique comments contributed by 3987 users. Seven sentiment models and one ensemble were evaluated, with RoBERTa-based transformer models selected for contextual analysis. Topic modeling was conducted using Latent Dirichlet Allocation and BERTopic. While BERTopic initially generated highly granular clusters, hierarchical merging produced stable, clinically interpretable themes. Six dominant discussion themes were identified: (i) autoimmune diagnosis and systemic symptoms; (ii) organ-specific concerns; (iii) fatigue, mobility, and sleep; (iv) navigating care pathways; (v) life impact; and (vi) coping strategies, lifestyle changes, and anxiety. Topic-linked sentiment analysis showed predominantly neutral sentiment overall. More negative sentiment was associated with unresolved symptoms and diagnostic uncertainty, and sentiment became increasingly negative at deeper comment levels, suggesting escalation toward unmet needs. Highly active users mainly contributed neutral, informational content. Temporal analysis identified episodic increases in engagement, including a peak in mid-2024 linked to discussions involving dermatomyositis, COVID-19, and treatment decisions. These findings demonstrate the feasibility of scalable NLP analysis of rare-disease online discourse and highlight how topic-linked emotional signals may indicate periods of uncertainty or emerging concerns.

## Introduction

Living with a long-term medical condition affects both physical and mental wellbeing, often creating uncertainty about symptoms, lifestyle impact, and self-management. Many individuals seek health information online to address these concerns (Wang and Cohen 2023, Rong et al. 2025).

Fox and Duggan (2013) reported that 35% of US adults have used the Internet to identify a potential medical condition. People with long-term medical conditions have been shown to use social media to share experiences, seek advice, and participate in peer-support communities, though the reliability of user-generated health information can remain a concern (Alhusseini et al. 2021, Shah 2024).

In healthcare data mining, Facebook and Reddit offer distinct analytical advantages due to differences in platform architecture and user engagement. Facebook emphasizes relationship-based interactions supported by rich multimedia formats, enabling analysis of patient networks, behavioral patterns, and longitudinal health engagement (Moorhead et al. 2013, van Dijck and Poell 2016, Qiao et al. 2024). Reddit, by contrast, provides a topic-centric and largely anonymous environment that facilitates open discussion of sensitive health issues, including addiction, mental illness, and long-term medical conditions (Baumgartner et al. 2020, Rajeev n.d.). These structural differences shape the types of insights each platform offers and influence their suitability for different forms of health-related research.

The large volumes of unstructured text generated on social media platforms present challenges for extracting clinically meaningful insights. Automated natural language processing (NLP) and machine learning approaches are therefore increasingly used to identify patterns that would be difficult to analyze manually. NLP has been applied to examine patient perspectives in online feedback and reviews (Ferguson et al. 2021, Khanbhai et al. 2021), including sentiment dynamics in cancer support forums and rheumatology-related Reddit discussions (Balakrishnan et al. 2021, García and Barbancho 2024). In rheumatoid arthritis research, NLP has been used to identify treatment experiences and symptom-related concerns (Kerkhof 2023) and to explore patient perceptions of disease-modifying anti-rheumatic drugs (Sharma et al. 2020). Beyond physical disease impacts, studies have also characterized stress-related expressions and temporal emotional patterns in subreddit communities (Tuan et al. 2024, Mansoor and Ansari 2024). Despite these advances, NLP remains challenged by linguistic variability in social-media text, including slang, abbreviations, emojis, and sarcasm, which can affect sentiment classification and topic extraction.

Facebook restricts access to group content through its Graph API, requiring formal approvals that limit data availability for observational studies. Reddit, by contrast, provides public access to anonymized discussions, enabling ethical data collection in line with established guidance for social media research (Ford et al. 2021, Harris et al. 2024).

Research examining social media use in rare diseases is limited (Boy et al. 2025), including myositis, a long-term muscle disorder characterized by weakness, fatigue, and systemic complication (Lundberg et al. 2018, Khoo et al. 2023). Myositis affects a relatively small population, with prevalence estimates ranging from a few to several tens of cases per 100 000 individuals (Khoo et al. 2023). Greater understanding of patient experiences may support earlier recognition of difficulties and improve supportive care.

This study conducts an exploratory analysis of myositis-related Reddit discussions to identify key themes, sentiment patterns, and engagement behaviors. Although the volume of online discourse is medium-sized, the analytic framework demonstrates how NLP methods can provide meaningful insight in rare disease contexts and may be generalizable to other less common medical conditions.

## Methods 

Ethical approval to conduct this work was granted by Birmingham City University, UK. Only publicly available posts and comments were analysed. Any personal identifiers including external hyperlinks were excluded from the dataset to maintain privacy. The study adhered to Reddit’s API terms of service and ethical research practices.

### Pre-processing

Data were extracted from publicly available Reddit discussions using the Python Reddit API Wrapper and pre-processed with the Natural Language Toolkit. Only English-language posts were included, and irrelevant or duplicate content was removed.

Seven established sentiment analysis models and one ensemble approach were evaluated on a de-duplicated corpus comprising 1223 unique posts and 18 453 unique comments. Lexicon in the form of latent Dirichlet allocation (LDA) and transformer (BERTopic) based analyses used the cleaned post and comment text fields to which lemmatization and token filtering were applied.

To perform the temporal analysis, a wider analytical pipeline was applied to the full Reddit dataset, in which each row represents a post–comment pair. Posts and comments are stored in a row-level structure that yields a dataset larger than the number of unique posts or unique comments. Following text cleaning, date-based filtering, and de-duplication of combined post–comment text for analyses 19 091 analytic rows were produced. These counts differ from the previous ones used for sentiment modeling due to the different units of analysis: row-level post–comment pairs for temporal and co-term analyses, whereas unique post or comment records were used for sentiment classification.

### Sentiment models

Two groups of sentiment models were used: lexicon-based and transformer-based (Table 1). Lexicon-based models assign polarity scores using predefined dictionaries and provide interpretable baselines, whereas transformer-based models capture contextual sentiment beyond surface-level word associations. All transformer models were applied without task-specific fine-tuning to preserve reproducibility and generalizability. BioClinicalBERT is not sentiment-tuned, so its outputs were interpreted heuristically. Together, the selected models were used to balance interpretability, contextual sensitivity, and applicability to health-related social media text.

**Table 1:** Sentiment models used in analysis.

Model	Category	Output type	Polarity scale	Training domain	Primary use
VADER	Lexicon	Continuous	−1 to +1	Social media	Baseline sentiment
TextBlob	Lexicon	Continuous	−1 to +1	General	Baseline comparator
AFINN	Lexicon	Integer (normalized)	Normalized	General	Cross-model comparison
Lexicon ensemble	Lexicon	Continuous	Z-scored	Combined	Stable lexicon estimate
RoBERTa-S (SieBERT)	Transformer	Label + probability	Signed polarity	General	Topic-linked sentiment
RoBERTa-C (CardiffNLP)	Transformer	Label + probability	Signed polarity + neutral	Social media	Conversational tone
DistilBERT Emotion	Transformer	Emotion labels	Valence-mapped	General	Emotion comparison
BioClinicalBERT	Transformer	Heuristic	Binary polarity	Clinical	Domain comparison

### Application and comparison strategy

To avoid bias from repeated posts or comments, sentiment was computed once per unique cleaned text. Each model was applied a single time to each unique post or comment, and outputs were then mapped back to the full dataset so that duplicate entries received identical sentiment values. All model comparisons were therefore conducted at the unique-text level.

Signed polarity values were compiled separately for unique posts and comments. Model agreement was assessed using Pearson correlation, Spearman correlation, and mean absolute error, alongside summary statistics including mean polarity and average cross-model agreement.

For analyses integrating sentiment with topic modeling (e.g. linking polarity to LDA-derived themes or extracting extreme polarity examples), sentiment values were derived using the RoBERTa-S model, which provides continuous polarity scores suitable for fine-grained comparison. For analyses of conversational tone and affective distribution across contributors, the RoBERTa-C model was used, as its explicit neutral category yields a more conservative distribution appropriate for distinguishing informational from affective communication.

### Statistical analysis

Sentiment differences across topics were analysed using the Kruskal–Wallis test, with eta-squared as effect size. Pairwise differences were assessed using Dunn’s post-hoc tests with Holm correction. Cliff’s delta quantified non-parametric effect sizes between topic pairs. Data in text is presented ±SD.

### Sentiment flow across comment depth

Sentiment flow (RoBERTa-S) was analysed across comment depth to characterize changes in emotional tone within discussion threads. Depth-specific mean sentiment, sentiment proportions, and sequential transition patterns were computed, with trends visualized using locally estimated scatterplot smoothing (LoESS). Full methodological details are provided in Supplementary Methods S1.

### Illustrative comment selection

To contextualize quantitative sentiment patterns, representative comments were extracted programmatically using sentiment scores without manual selection. These comments were not subjected to qualitative analysis and were used solely to illustrate model-predicted sentiment extremes across predefined comment-depth bands. Full selection logic and eligibility criteria are described in Supplementary Methods S4.

### Key word analysis

For keyword extraction and topic modeling, text was filtered using a combination of standard stopwords, custom domain-neutral terms, and part-of-speech (POS) constraints to emphasize clinically meaningful language. High-frequency generic verbs and conversational fillers were explicitly removed to reduce noise; alongside custom terms commonly used in informal discussion but lacking clinical specificity.

To preserve clinically relevant action terms, a predefined set of medical and functional verbs (e.g. diagnose, test, walk, move, struggle) was explicitly retained. POS filtering was applied to keep nouns and adjectives, together with the retained clinical verbs, ensuring emphasis on symptoms, diagnostic processes, body regions, and treatment-related terminology.

Complete lists of removed and retained terms are provided in Supplementary Methods S5 to support reproducibility while maintaining clarity in the main Methods description.

### Top-word frequency analysis

Following preprocessing, lemmas were extracted separately for posts and comments. Tokens were flattened and frequency-counted, and the top 30 terms were identified for each. These terms were used to generate bar plots representing dominant clinical language across the corpus.

### Next-layer keyword analysis

To examine secondary concerns beyond explicit disease framing, a predefined set of disease- and diagnosis-related anchor terms (e.g. disease, diagnosis, autoimmune, rheumatologist, test, lab) was removed from the deduplicated topic corpus. The remaining tokens were grouped into four categories based on clinical relevance: symptoms, diagnostic challenges, treatment concerns, and psychosocial impacts. The most frequent terms within each category were summarized and visualized using horizontal bar charts to illustrate their relative prominence.

### Analysis of top contributors: lexical patterns, sentiment, and temporal activity

To characterize the clinical focus of the most active users, keyword extraction pipeline was repeated on a subset comprising comments from the top ten contributors. The same lemmatization and frequency-based keyword approach was used, with an expanded domain-specific stopword list to remove generic conversational tokens e.g. “like, sorry, maybe, think” and low-value lexical items. Clinically relevant terms, for example “diagnose, test, walk, move, struggle” were explicitly retained. Output was restricted to the top 35 terms to maximize interpretability and to reflect the highly clinical nature of discussions among this subset of users. In addition, a transformer-based sentiment classifier (RoBERTa-S) was applied to all comments from these contributors to quantify the emotional tone of their discourse.

A temporal activity analysis was performed to examine patterns of community engagement over time. Timestamps associated with each comment were converted to monthly periods, covering the dataset time range from January 2020 to November 2024. A truncated timeline was generated beginning at the point where monthly contributor counts first exceeded a threshold identified through exploratory analysis (≥200 contributors), in order to focus on periods of sustained activity. For each month, the number of unique contributors was calculated to quantify fluctuations in participation. These monthly profiles were used to characterize changes in participation volume and to identify periods of increasing, peak, and declining engagement across the dataset.

### Topic modeling

LDA and BERTopic were used to identify discussion themes. Prior to topic modeling, frequent multi-word expressions were identified using collocation-based phrase detection (Gensim Phrases) to characterize common bigrams in the corpus. Topic models were applied to a de-duplicated corpus of lemmatized text. LDA was implemented with six topics, with document–topic probability distributions used to assign dominant themes. BERTopic was applied to the same corpus to generate base topics, which were subsequently clustered into six higher-level super-topics using hierarchical clustering to enable direct comparison with the six-topic LDA solution. Detailed BERTopic configuration and topic-merging procedures are provided in Supplementary Methods S2.

### Topic coherence

Topic coherence for LDA was evaluated on the de-duplicated corpus using Gensim’s coherence model, computing both c_v and u_mass coherence. Coherence was calculated using the top 10 words per topic extracted from the fitted LDA model.

### BERTopic modeling and sentiment analysis

BERTopic was applied to the same lemmatized corpus used for LDA to identify contextual topic structure. Base topics and an outlier class were generated and subsequently merged into six super-topics via hierarchical clustering to enable direct comparison with the six-topic LDA solution. Super-topics were aligned to LDA themes by assigning each cluster the dominant LDA topic label among its member documents, while outlier posts were retained separately. Full clustering configuration and alignment procedures are detailed in Supplementary Methods S2.

Sentiment scores were derived using the RoBERTa-S classifier and analysed across BERTopic super-topics. Sentiment labels were mapped back to cleaned comments to support temporal and role-based analyses. Between-topic sentiment differences were assessed using Kruskal–Wallis tests with Dunn’s post-hoc comparisons and Holm correction. Cross-model sentiment associations were evaluated at the document level and using aligned topic labels. Variance in sentiment attributable to topic structure was summarized using ordinary least squares models; full regression specifications are provided in Supplementary Methods S3.

### Top contributors, role, and sentiment

To characterize conversational behavior among the ten most active contributors, each unique cleaned comment was classified into one of three categories: help-seeking, support-offering, or neutral, using a rule-based pattern-matching approach. Comments were labeled as support-offering when they contained predefined supportive phrases indicating reassurance or encouragement (e.g. “stay strong”, “here for you”). Help-seeking comments were identified using predefined enquiry-based patterns or, where no explicit pattern was matched, the presence of a question mark (e.g. “should I raise this with my doctor?”). Comments that matched neither rule set were labeled as neutral. Where both support-offering and help-seeking patterns were present, support-offering labels were prioritized. Classification was performed on de-duplicated comments, and category distributions were summarized for the top ten contributors to assess variation in discourse style.

The RoBERTa-C sentiment model was then applied to comments from the same ten contributors, using the de-duplicated dataset (2020 onwards). For each author, the pipeline returned sentiment labels (positive, neutral, negative), confidence scores, overall sentiment distributions, author-specific sentiment proportions, and representative example comments for each sentiment class.

### Temporal analysis

To align analyses with the growth of the Reddit community, all rows with comment timestamps before 1 January 2020 were excluded. A predefined clinical lexicon was applied for temporal and correlation analyses, comprising terms related to dermatomyositis, myositis-specific antibodies, inflammatory markers, symptoms, treatments, respiratory involvement, and infectious triggers. Example terms included “dermatomyositis, myositis, weakness, rash, dysphagia; antibodies (Mi-2, SRP, Jo-1)”; markers (CK, CRP, ESR); treatments (IVIG, methotrexate, rituximab, mycophenolate); respiratory terms (lung, interstitial); and infectious triggers (COVID, viral, infection). Only terms occurring at least once between 2020 and 2024 were retained.

Monthly frequencies of all lexicon terms were calculated from merged post–comment text. Term counts from April to August 2024 were compared with a 12-month baseline period (April 2023–March 2024) using a “spike ratio” defined as the mid-2024 count divided by the baseline count, with baseline values of zero set to one to avoid division inflation. Ratios greater than one were interpreted as temporal increases. In addition, monthly term frequencies from January 2020 to November 2024 were used to compute Pearson correlations between each term and “dermatomyositis”, with terms ranked by correlation coefficient to identify those most strongly co-varying over time. A summary of the datasets used is presented in Table 2

**Table 2:** Mapping of analytical tasks to data units.

Analysis	Unit
Temporal trends	Post–comment rows
Sentiment distribution	Unique cleaned texts
Top-contributor characterization	Unique cleaned comments
Topic modeling	Document-level text

#### Data visualization

Matplotlib was used for the creations of charts, histograms, and word clouds.

## Results

The study was conducted in 2024. The dataset comprised 1223 unique posts and 18 453 unique comments contributed by 3987 users collected between 2016 and 2024. For temporal and trend analyses, an analytical time filter focusing on the period 2020–2024 was applied, reflecting higher volumes of posting activity during this interval.

### Sentiment analysis

An initial model consistency check was conducted using the lexicon-based tools VADER and TextBlob, which showed moderate correlation (posts: r = 0.42; comments: r = 0.43). Combining these models into a lexicon ensemble improved internal stability, with stronger correlations observed against both VADER (posts: r = 0.80; comments: r = 0.83) and TextBlob (posts: r = 0.84; comments: r = 0.85). This indicates that the ensemble captures shared polarity signals while reducing model-specific noise, making it the most stable non-transformer sentiment estimator. Sentiment outputs were subsequently evaluated across all eight models at the unique-text level, with de-duplicated predictions mapped back to the full dataset to ensure complete model coverage and no missing data (Supplementary Table S1).

While the lexicon ensemble improved stability relative to individual lexicon models, transformer-based sentiment analysis demonstrated greater contextual sensitivity, making it more appropriate for semantic interpretation alongside topic modeling. Other models that were evaluated included AFINN, DistilBERT Emotion, and individually VADER or TextBlob. These produced valid outputs but showed weaker agreement and higher error rates than the selected ensemble and RoBERTa models. BioClinicalBERT, despite being domain-specific demonstrated limited suitability for sentiment analysis due to its restricted score range and reliance on heuristic polarity assignment, resulting in low variability and reduced interpretability.

**Figure 1:**
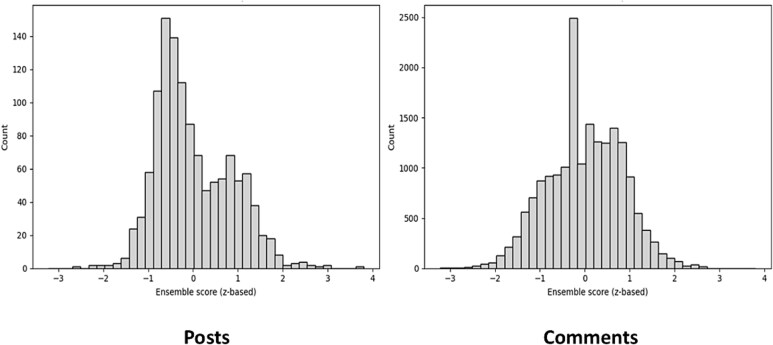
Sentiment distribution in myositis subreddit posts and comments. The *y*-axis scales differ between panels due to the higher volume of comments relative to posts; separate scaling was retained to preserve detail in the post distribution while allowing comparison of overall sentiment trends.

The lexicon ensemble produced sentiment distributions centered near zero for both posts and comments (mean = 0.00 for both) (Fig. 1). Posts showed a slightly negative skew (median −0.19), reflecting symptoms and diagnostic reporting, while comments were near neutral (median −0.00), indicating more balanced or supportive responses.

This indicates that discourse on the subreddit is broadly neutral with predictable emotional variation driven by symptom reports and community replies.

### Sentiment flow by comment depth

**Figure 2:**
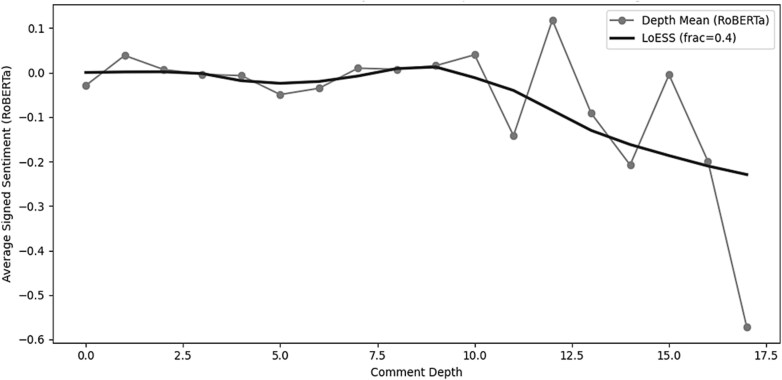
Average RoBERTa-S based signed sentiment by comment depth, including depth-level means and LoESS-smoothed trend.

The LoESS-smoothed sentiment curve showed a consistent relationship between comment depth and emotional tone (Fig. 2).

Table 3 shows the condensed sentiment metrics by comment depth and summarizes the main quantitative patterns that underpin the LoESS curve.

**Table 3:** Condensed sentiment metrics by comment depth.

Depth	Comments (*N*)	Unique (*N*)	Positive (%)	Negative (%)	Mean sentiment (±SD)
0	9768	7943	48.5%	51.5%	−0.029 ± 0.988
1	5894	4645	52.0%	48.0%	+0.038 ± 0.990
2	3437	2707	50.4%	49.6%	+0.007 ± 0.989
3–5	3550	2761	49.2%	50.8%	−0.013 ± 0.989
6–10	1026	752	49.7%	50.3%	−0.005 ± 0.990
11+	150	95	45.3%	54.7%	−0.093 ± 0.993

Shallow depths were near neutral, mid-depth replies remained stable, and the deepest threads showed a shift toward more negative sentiment. Although these depths contained fewer comments, contributing to greater variability in raw values, the LoESS trend consistently indicated increasingly negative sentiment. Overall, the LoESS curve shows a neutral-to-positive start, stable mid-depth neutrality, and progressively negative sentiment in the deepest thread segments.

To illustrate the quantitative sentiment patterns across comment depth, example comments with the strongest positive and negative RoBERTa-S sentiment scores were extracted for each depth band.

At shallow depths (0–2), comments reflected a balance of gratitude and frustration, consistent with near-neutral mean sentiment. Strongly positive examples expressed appreciation (positive, depth 1, sentiment +0.999: “I will definitely check this out, thank you so…”), while negative examples conveyed dissatisfaction with care (negative, depth 1, sentiment −0.999: “I do agree. I feel very let down by this doctor…”).

Mid-depth comments (3–5) similarly contained both supportive and dissatisfied responses. Positive examples included acknowledgment and reassurance (positive, depth 3, sentiment +0.999: “I appreciate the rational explanation in your …”), whereas negative comments reflected more detailed frustration with healthcare interactions (negative, depth 3, sentiment −0.999: “It feels even worse coming from medical professionals…”).

At mid–late depths (6–10), comments continued to span gratitude and disagreement, illustrating the coexistence of supportive and disputative interaction within extended exchanges (e.g. positive, depth 7, sentiment +0.999: “I’ve been sent there through the VA’s Community…”; negative, depth 10, sentiment −0.999: “Still waiting for your sources… You claim to be…”).

At the deepest levels (11+), comments showed a shift toward more confrontational or distressed language. While supportive messages remained present (positive, depth 11, sentiment +0.999: “Thank you. I definitely feel more empowered…”), strongly negative exchanges were more frequent (negative, depth 14, sentiment −0.999: “Is this comment made just to insult me?”), aligning with the increasingly negative sentiment observed at greater comment depth.

### Top-word frequency analysis

**Figure 3:**
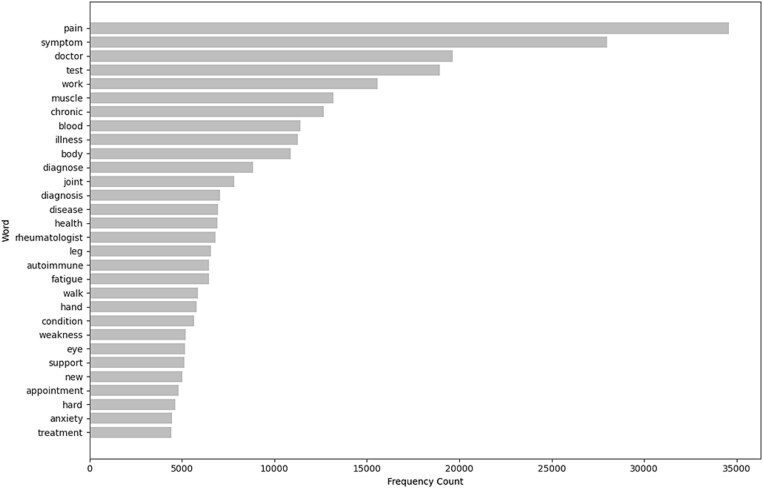
Top-ranked words by frequency within posts and comment.

Analysis of the de-duplicated, clinically filtered corpus showed that discussions were dominated by core symptom and diagnostic terminology (Fig. 3). Terms relating to pain, symptoms, medical consultation, and testing were most frequent, followed by words reflecting functional limitation and chronic disease burden. Diagnostic and anatomical concepts were also strongly represented, while psychosocial terms such as support and anxiety appeared less frequently. Overall, the distribution highlights discourse centered on symptom burden, diagnostic navigation, and functional impact

### Next-layer keyword analysis

**Figure 4:**
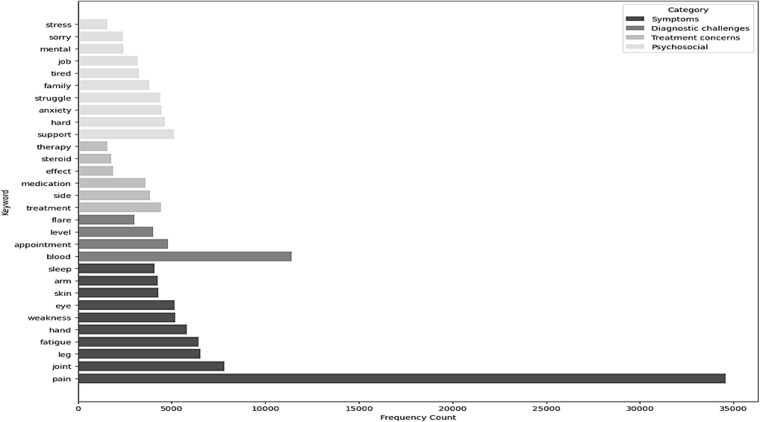
Most frequent keywords organized by thematic category, showing relative occurrence within posts and comments.

After removing explicit disease terminology, a secondary layer of clinically interpretable terms emerged (Fig. 4). Symptoms remained the dominant category, with pain continuing to feature most prominently, followed by terms relating to joint involvement, fatigue, limb weakness, sensory disturbance, and sleep disruption. Diagnostic challenges formed the next most prominent group, including references to laboratory monitoring, clinical appointments, disease flares, and fluctuating biomarker levels, indicating frequent discussion of diagnostic uncertainty and ongoing clinical surveillance. Psychosocial terms appeared with moderate frequency and reflected emotional burden and social impact, including expressions of anxiety, struggle, fatigue, work-related concerns, and family roles. Treatment-related terms were less frequent encompassing medications, side effects, and therapeutic decision-making. Together, these distributions highlight symptom burden and diagnostic navigation as dominant concerns, with psychosocial and treatment issues forming secondary but persistent themes.

### Topic modeling

LDA was applied to the de-duplicated corpus. By the inclusion of top words and statistically meaningful bigrams for example “nerve pain, doctor appointment, muscle weakness” improved separation of symptom-based and functional impact themes, leading to clear differentiation into symptom clusters, psychosocial concerns, and healthcare navigation issues which were then categorized into six coherent and clinically interpretable topics:

1) Autoimmune diagnosis, limb weakness, and systemic symptoms;
2) Localized organ symptoms (chest, skin, vision);
3) Physical weakness, fatigue, sleep, and daily functioning;
4) Healthcare navigation, appointments, and treatment pathways;
5) Functional disability, life impact, and social roles; and
6) Anxiety, diet, somatic concerns, and coping behaviors.

### Topic model coherence for latent Dirichlet allocation

To assess internal consistency and interpretability of the six-topic LDA model, topic coherence was evaluated using *c_v* and *u_mass* metrics. Across the de-duplicated corpus, the model achieved an overall *c_v* coherence of 0.468 and *u_mass* coherence of −1.297, indicating moderate semantic coherence typical of short, informal health-related social media text. Topic-level results showed variation in coherence across themes. Topic 1 (autoimmune diagnosis, limb weakness, and systemic symptoms) demonstrated the highest coherence (*c_v* = 0.537; *u_mass* = −0.999), indicating a well-defined semantic cluster. Topics related to localized symptoms (Topic 2; *c_v* = 0.489) and functional disability and life impact (Topic 5; *c_v* = 0.486) also exhibited relatively strong coherence.

Lower coherence was observed for more heterogeneous themes. Topic 6 (anxiety, diet, somatic concerns, and coping behaviors) showed the lowest coherence (*c_v* = 0.422; *u_mass* = −1.522), consistent with its broad thematic scope, while Topic 4 (healthcare navigation, appointments, and treatment pathways) exhibited moderate coherence (*c_v* = 0.434). Inclusion of bigrams improved semantic granularity and interpretability, enabling clearer separation of symptom-based, healthcare navigation, and psychosocial themes. Overall, these results indicate that the LDA model produced interpretable topic structures appropriate for the complexity and variability of patient-generated discourse.

### Sentiment analysis of latent Dirichlet allocation topics

Sentiment was extracted using RoBERTa-S and matched to each document’s dominant topic. Polarity scores revealed substantial variation in emotional tone across topics. Topics 1–5 had negative sentiment, indicating distress, uncertainty, pain, or frustration. Topic 2 (localized organ symptoms) was the most negative. Topic 6 (anxiety, diet, and coping) was the only positive topic, reflecting coping and support narratives (Table 4).

**Table 4:** Mean sentiment polarity.

Topic	Mean polarity (±SD)	*N*
1. Autoimmune diagnosis, limb weakness and systemic symptoms	–0.513 ± 0.848	9648
2. Localized organ symptoms (chest, skin, vision)	–0.721 ± 0.663	1529
3. Physical weakness, fatigue, sleep and daily functioning	–0.638 ± 0.757	1938
4. Healthcare navigation, appointments and treatment pathways	–0.305 ± 0.938	3470
5. Functional disability, life impact and social roles	–0.277 ± 0.956	3358
6. Anxiety, diet, somatic concerns and coping behaviors	+0.186 ± 0.924	2581

### Statistical analysis

Kruskal–Wallis (H = 508.9, *P*< 1 × 10^−100^, ε^2^ = 0.02). Dunn post-hoc (Holm corrected). Pairwise comparisons were statistically significant, particularly between Topic 6 (positive) versus all negative topics. Topic 2 (most negative) vs. Topics 4–6. Cliff’s Delta showed strong contrasts between Topic 6 and Topics 1–3 (δ = 0.26–0.35) and moderate differences between distress-heavy topics (1–3) and coping-oriented topics (4–6).

### Topic-level sentiment (latent Dirichlet allocation) illustrated with indicative comments

To contextualize topic–sentiment patterns, exemplar comments were extracted programmatically using RoBERTa-S sentiment polarity, including the most positive and most negative comments within each topic. Additional representative comments were sampled to illustrate typical content. These excerpts were used solely for illustration and were not analysed qualitatively.

#### Autoimmune diagnosis, limb weakness, and systemic symptoms

This topic showed consistently negative sentiment. The most negative comments expressed explicit frustration with healthcare interactions, while the most positive described constructive clinical engagement, including rheumatological evaluation for suspected dermatomyositis. Representative comments near the mean polarity (−0.576) described persistent muscle pain, weakness, and fatigue, alongside difficulty articulating gradual functional decline.

#### Localized organ symptoms (chest, skin, vision)

This was the most negatively polarized topic. Highly negative comments reflected anxiety related to vision impairment and impending clinical appointments, while positive-classified comments still described substantial disease burden (e.g. disabling nerve pain), likely reflecting explanatory or treatment-oriented framing. Representative comments (mean −0.647) described recurrent, patterned progression of organ-specific symptoms, including respiratory and visual involvement.

#### Physical weakness, fatigue, sleep, and daily functioning

Negative exemplars described feeling unsafe at work, mistrust of medical care, and worsening pain, often repeated across posts. Positive exemplars focused on practical adaptations or treatment changes (e.g. assistive devices, medication adjustments). Representative comments near the mean polarity (−0.638) emphasized longstanding functional impairment, progressive leg pain, and concern about irreversible damage without timely diagnosis.

#### Healthcare navigation, appointments, and treatment pathways

Negative comments highlighted frustrating clinical encounters and dissatisfaction with care pathways, while positive examples reflected community encouragement and support-oriented messages. Representative comments (mean −0.576) captured the cumulative burden of slow diagnostic processes and ambivalence about illness disclosure, illustrating tension between care navigation and identity management.

#### Functional disability, life impact, and social roles

Negative exemplars centered on limited familial support and distress related to abnormal laboratory findings and perceived medical inaction. Positive exemplars emphasized psychological support, peer connection, and sustained engagement with care. Representative comments (−0.650 to −0.711) described ongoing functional limitation despite partial symptom control, reflecting mixed but predominantly negative tone.

#### Anxiety, diet, somatic concerns, and coping behaviors

This was the only topic with a positive mean polarity. Negative comments reflected relational strain and lack of symptom validation, whereas positive ones focused on adaptive coping strategies, including lifestyle modifications and symptom management. Representative comments near the mean polarity (≈0.58) described somatic symptoms (e.g. dizziness, severe episodes) in a pragmatic, matter-of-fact manner consistent with a coping-oriented framing.

Full sets of topic-specific exemplar comments, including polarity values and depth metadata, were generated programmatically and are summarized in Supplementary Results S2.

### BERTopic and sentiment

BERTopic modeling generated 28 base clusters, including an outlier class. Inspection of top-ranked terms indicated that many base clusters reflected narrow lexical themes, limiting clinical interpretability compared with the LDA solution. To address this, hierarchical topic reduction was applied, yielding six clinically coherent super-topics:

- Super-topic 1: anxiety, diet, somatic concerns, and coping behaviors (mean sentiment 0.989 ± 0.040; *n* = 2606);
- Super-topic 2: autoimmune diagnosis, limb weakness, and systemic symptoms (0.989 ± 0.037; *n =* 4671);
- Super-topic 3: functional disability, life impact and social roles (0.990 ± 0.036; *n =* 469);
- Super-topic 4: autoimmune diagnosis, limb weakness, and symptom progression (0.991 ± 0.033; *n =* 170);
- Super-topic 5: anxiety, diet, somatic concerns, and coping behaviors (secondary cluster) (0.988 ± 0.043; *n =* 443); and
- Super-topic 6: physical weakness, fatigue, sleep, and daily functioning (0.990 ± 0.040; *n =* 521).

Two super-topics captured autoimmune-related discourse: one characterized by prolonged symptom burden and diagnostic uncertainty, and the other by clearer symptom progression and escalation, aligning with the autoimmune pathways identified in the LDA model. Overall, BERTopic super-topics mapped closely onto the same clinical and psychosocial structure identified by LDA, including autoimmune diagnosis and systemic symptoms, functional disability and life impact, and anxiety- and coping-oriented narratives.

BERTopic sentiment outputs should not be interpreted as absolute emotional polarity. RoBERTa-C sentiment scores are probability-scaled and constrained to an upper-range distribution without negative values. Consequently, observed differences represent comparative variation between clusters rather than absolute shifts in emotional tone.

Statistical testing identified significant sentiment differences across both LDA topics (Kruskal–Wallis H = 213.7, *P* < 1 × 10^−43^) and BERTopic super-topics (H = 75.1, *P* < 1 × 10^−13^). Dunn post-hoc comparisons confirmed multiple significant pairwise contrasts, although effect sizes were small, consistent with the compressed sentiment range. Topic-level sentiment correlation between LDA and BERTopic was moderate (r = 0.582), while correlation at the aligned-label level was lower (r = 0.220). Document-level correlation was 1.0, reflecting use of the same underlying sentiment scores. Across both models, sentiment *z*-scores showed minimal deviation from zero (~−0.30 to +0.30), indicating limited clustering of extreme emotional tone.

Representative posts for each BERTopic super-topic were selected using RoBERTa-S sentiment probability scores (0–1 scale). For each super-topic, the lowest-, highest-, and mean-proximal sentiment posts were identified, mirroring the LDA exemplar selection procedure while accounting for differences between probability-based and polarity-based sentiment outputs.

#### Super-topic 1: autoimmune diagnosis, limb weakness, and systemic symptoms

Aligned primarily with LDA Topic 1, this super-topic captured broad systemic disease narratives. The most negative comments (polarity ≈ −1.000) described distress related to worsening symptoms, including difficulty walking, muscle weakness, and repeated clinical appointments. The most positive posts (polarity ≈ 0.999) emphasized meaningful clinical progress, such as referrals, successful consultations, or treatment optimization. Representative posts (polarity ≈ −0.576) described gradual deterioration and worsening muscle fatigue (e.g. “mustering energy”), closely reflecting a systemic myositis-like presentation.

#### Super-topic 2: functional disability, life impact, and social roles

This super-topic mapped closely to LDA Topic 5. The most negative comments (polarity ≈ −1.000) focused on feeling dismissed regarding hormonal abnormalities (e.g. prolactin or thyroid), inability to work, and restricted daily functioning. In contrast, the most positive examples (polarity ≈ 0.999) highlighted sustained therapeutic progress, psychological support, or improved access to services. Representative posts (polarity ≈ −0.650) described decisions around disclosure, altered life plans, and identity adaptation in response to chronic illness.

#### Super-topic 3: autoimmune diagnosis, limb weakness, and symptom progression

This super-topic represented a narrower subset of autoimmune progression narratives. The most negative content (polarity ≈ −1.000) described repeated dismissal, multisystem flares, and escalation in organ involvement, whereas the most positive content (polarity ≈ 0.999) reflected diagnostic clarity or effective clinician-led investigation. Representative posts (polarity ≈ −0.576) described slow but recognizable deterioration, often involving respiratory, visual, or neuromuscular changes. Compared with super-topic 1, this group was distinguished by clearer temporal sequencing and stronger emphasis on escalation milestones.

#### Super-topic 4: anxiety, diet, somatic concerns, and coping behaviors

Aligned with LDA Topic 6, this super-topic showed weaker emotional polarity overall. The most negative posts (polarity ≈ −1.000) reflected relational tension, including parental invalidation of symptoms and anxiety around self-management. The most positive content (polarity ≈ 0.999) consisted of requests for coping strategies, such as equipment use, supportive bedding, or dietary changes. Representative posts (polarity ≈ 0.576) described somatic experiences including dizziness, nausea, and weakness, conveyed in a practical or neutral tone rather than overt distress.

### Top contributors

**Figure 5:**
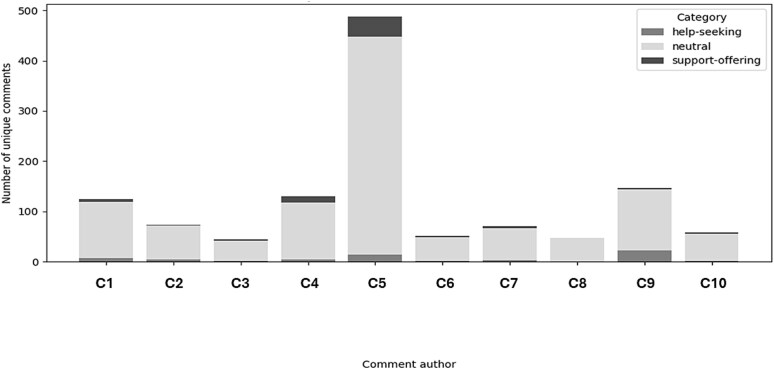
Bar chart showing the proportion of help-seeking, support-offering, and neutral posts by the top ten contributors, with neutral posts dominating..

Contributions were further examined by focusing on the most frequent posters. The ten most active contributors exhibited a mix of help-seeking, support-offering, and neutral roles, with neutral posts predominating particularly among contributors who primarily asked questions or shared general observations (Fig. 5).

### Time based trends

**Figure 6:**
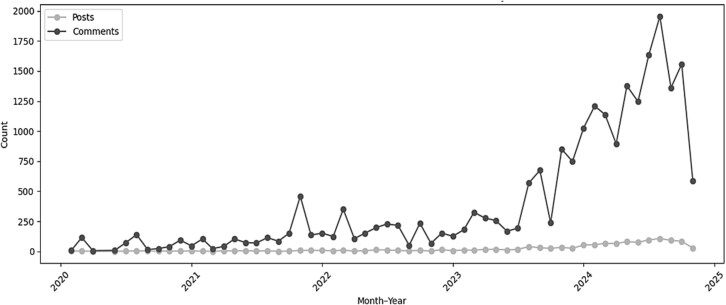
Monthly counts of posts and comments from 2020–2024.

Analysis of user activity in the myositis subreddit between the years 2016–2024 showed the volume of activity increased steadily from 2020 to late 2023, followed by a pronounced rise in early mid-2024 (Fig. 6). Posts per month remained relatively stable across the 2020–2024 window. In contrast, comments increased sharply, particularly from late 2022 onward and accelerating through 2023–2024.This resulted in substantial growth in community engagement without an increase in the number of initiating posts. The rise in comments suggests that existing posts attracted more discussion, with conversations becoming increasingly active and extended over time.

To characterize informational dynamics, the ten most active contributors (C1–C10), ranked by unique comment count, were analysed. Across these users, most comments were classified as neutral and typically consisted of informational statements, diagnostic interpretation, or descriptions of personal clinical experience. Neutral comments, such as C6 “did you have a myositis panel?”, accounted for 83%–100% of each contributor’s output, indicating that highly active users primarily engage in information exchange rather than affective communication.

Help-seeking comments, for example C8 “What happens if it is myositis and I don’t get treated”, involved clarification regarding symptoms, diagnostic procedures, or treatment effects and were less common (0%–15%). Support-offering comments, such as C7 “If I’m any kind of hope that really makes me feel better today so thanks for letting me know”, were comparatively rare (0%–9%), suggesting that while emotional reassurance occurs, it represents a minor component of engagement among high-activity users.

A domain-filtered lexical analysis of comments from these contributors revealed strongly clinical discourse. The 35 most frequent terms were dominated by symptoms, diagnostics, and specialist care pathways, including symptom (*n* = 503), autoimmune (*n* = 438), muscle (*n* = 377), disease (*n* = 344), test (*n* = 275), diagnosis (*n* = 249), doctor (*n* = 246), myositis (*n* = 240), and pain (*n* = 199). Diagnostic markers and procedures were also prominent (ANA *n* = 185, diagnose *n* = 178, antibody *n* = 143, biopsy *n* = 134, lab *n* = 130, blood *n* = 119, panel *n* = 75, testing *n* = 78), alongside references to specialist care (rheumatologist *n* = 160, rheum *n* = 150, treatment *n* = 154, treat *n* = 106, neurologist *n* = 71). Symptom and impairment terms such as rash (*n* = 113), immune (*n* = 102), skin (*n* = 98), weakness (*n* = 88), fatigue (*n* = 87), leg (*n* = 76), flare (*n* = 76), and damage (*n* = 66) reflected discussion of inflammatory manifestations and disease progression. The presence of COVID (*n* = 69) aligned with reports of symptom exacerbation or onset following SARS-CoV-2 infection.

Sentiment analysis using a RoBERTa-C classifier was applied to 1226 de-duplicated comments from the top ten contributors. Most comments were neutral (45.8%), followed by negative sentiment (44.5%), with positive sentiment comprising a smaller proportion (9.8%). Sentiment varied across contributors: negative sentiment was highest for C3 (58.1%) and C1 (51.6%), whereas C2 (73.0% neutral) and C6 (64.0% neutral) showed predominantly neutral interaction. Positive sentiment was proportionally highest for C8 (19.1%) and C5 (13.0%) but remained uncommon overall.

Across contributors, neutral sentiment aligned with symptom description, laboratory discussion, and clinical information exchange. Negative sentiment reflected diagnostic uncertainty, treatment limitations, and ongoing symptom burden, while positive sentiment was associated with encouragement, reassurance, or acknowledgement of progress. Overall, these findings indicate that highly active contributors primarily facilitate information-oriented discourse, playing a key role in helping others navigate complex clinical information.

**Figure 7:**
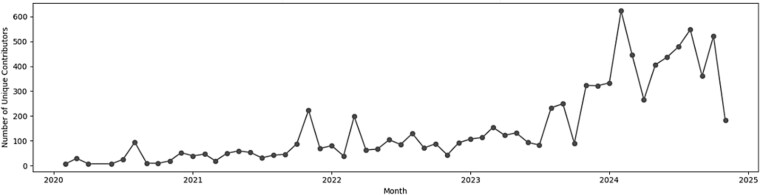
Monthly unique contributor counts from 2020–2024.

Analysis of monthly participation across the full dataset showed substantial variation in the number of unique contributors. Activity remained low and relatively stable in the early years, followed by a gradual increase starting from 2020, that accelerated during 2023 and 2024, indicating sustained growth in community engagement. Contributor numbers peaked in mid-2024 before declining slightly toward the end of the observation period in November 2024 (Fig. 7).

**Figure 8:**
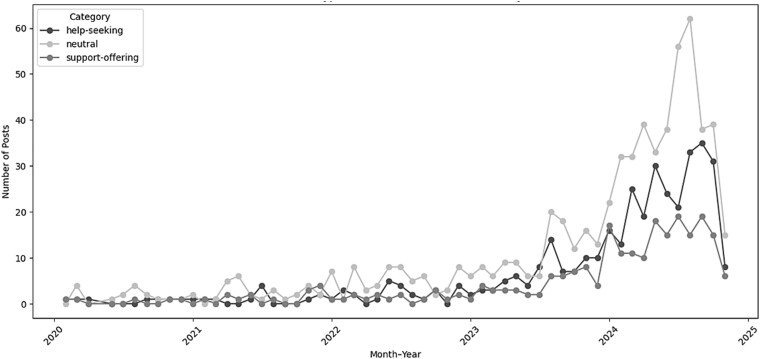
Trends in post types (help-seeking, support-offering, neutral) over time, showing neutral posts most common and support-offering consistently low..

When the posts were reviewed with reference to the three functional types (help-seeking, support-offering, and neutral), neutral posts which were predominantly composed of questions and/or observations were the most common during mid-2024 (Fig. 8).

Analysis of the mid-2024 temporal spike showed discussion concentrated around several clinically coherent categories. Treatment-related terms were prominent, including rituximab, methotrexate, mycophenolate, prednisone, prednisolone, IVIG, and steroids. Myositis-specific autoantibodies were also frequently discussed, most notably Mi-2 and SRP, with less frequent reference to antisynthetase antibodies. Laboratory and inflammatory markers such as CK, CRP, ESR and ANA were commonly mentioned.

**Figure 9:**
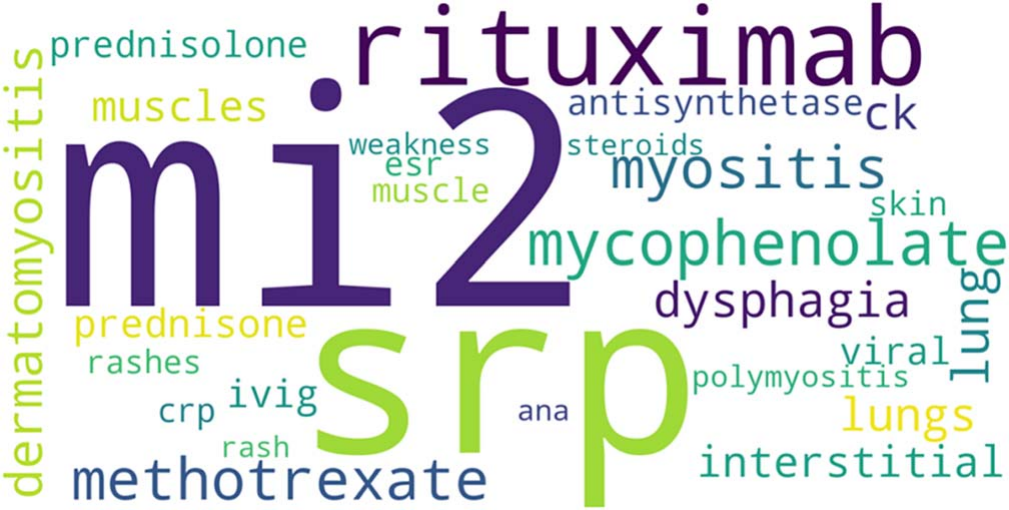
Word cloud showing dominant treatment, autoantibody, inflammatory, and symptom-related terms during mid-2024 myositis discussions.

Symptom-focused terms featured strongly (dysphagia, muscle, weakness and rash), alongside references to organ involvement, particularly pulmonary and cutaneous manifestations (lung, interstitial and skin), and viral contexts. Collectively, the word cloud (Fig. 9) indicates that the mid-2024 spike was dominated by treatment discussions, autoantibody profiles, and muscle- and lung-related manifestations consistent with dermatomyositis activity patterns.

**Figure 10:**
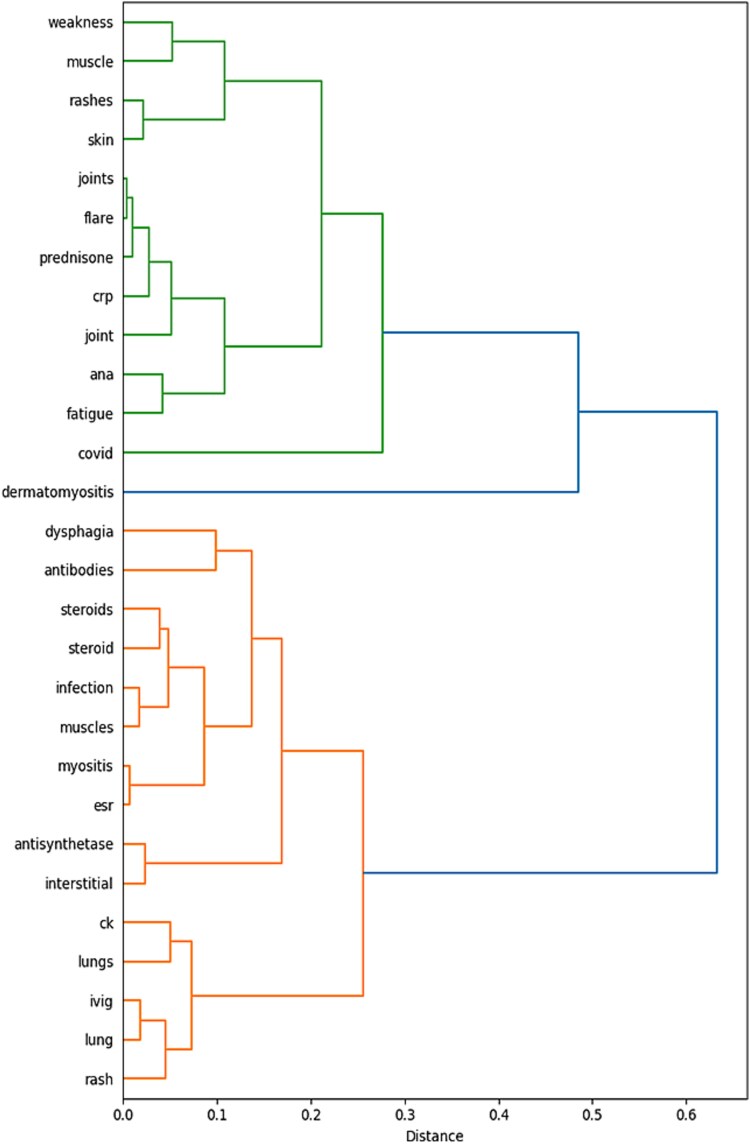
Hierarchical clustering of term–trend correlations for COVID-19 and dermatomyositis (2020–2024), revealing distinct clusters of treatment-related terms, diagnostic markers, and symptom or organ involvement. COVID-19 activity clusters closely with dermatomyositis and markers of systemic inflammation.

Hierarchical clustering of term–trend correlations showed that discussions around COVID-19 and dermatomyositis were embedded within broader, clinically coherent groups (Fig. 10). Treatment-related terms clustered together, including rituximab, methotrexate, mycophenolate, IVIG, and corticosteroids (prednisone, prednisolone). Myositis-specific antibodies (Mi-2, SRP, antisynthetase) and inflammatory markers (CK, ESR, CRP, ANA) formed a distinct diagnostic cluster. Symptom and organ-involvement terms such as lung(s), interstitial, dysphagia, rash, skin, muscle, and weakness grouped closely, reflecting the characteristic clinical manifestations of dermatomyositis. COVID-related temporal activity aligned most strongly with dermatomyositis and markers of systemic inflammation, consistent with the correlation analysis.

Overall, the clustering confirmed that COVID-19– and dermatomyositis-related discussions were not isolated phenomena but part of broader, clinically interpretable patterns spanning treatment decision-making, diagnostic markers, and multisystem involvement.

## Discussion

Social media provides a rich source of health-related information that can offer useful insights for the clinical community, including identification of key topics of interest and time-related events. This study demonstrates the feasibility of applying automated NLP techniques to rare disease discourse using a medium-sized dataset. The intention was not to generalize findings to the broader myositis population, but to establish a methodological foundation for future, larger-scale studies conducted over extended timeframes or across multiple platforms.

Reddit discussions relating to the long-term medical condition myositis were analysed due to the platform’s public accessibility and the presence of sustained, in-depth discussion not readily available on other platforms such as Facebook. Analyses of trends, engagement dynamics, and sentiment showed that users engage with the subreddit primarily as a space for information exchange, experiential sharing, and advocacy. At the time of the study, the community comprised nearly 4000 users. The subreddit served a dual role as both a knowledge-sharing environment and a source of emotional support, as reflected in topic modeling outputs. The lexicon-based LDA approach generated coherent clusters representing well-defined healthcare discussion structures, while the transformer-based BERTopic approach yielded comparable themes after hierarchical merging to enable clinically meaningful interpretation.

Identified themes encompassed symptoms, diagnostics, emotional experiences, and long-term healthcare strategies, with diagnostic procedures and clinical evaluation receiving the highest levels of contribution. Posts discussing diagnosis, treatment efficacy, and physical challenges were frequently associated with expressions of shared struggle or success, reinforcing the subreddit’s informational and psychosocial functions. These findings align with prior literature on long-term illness, which highlights the role of user-generated content in facilitating peer support and exchange of clinically relevant knowledge (Thompson et al. 2022). Concerns expressed by myositis patients in this study also parallel broader evidence that long-term systemic illness is associated with reduced quality of life and impaired work capacity (Anyfanti et al. 2016) with disease severity, fatigue, and mental health challenges contributing substantially to diminished well-being.

Overall emotional tone was predominantly neutral, with negative sentiment reflecting pain, frustration with healthcare systems, or the emotional burden of long-term illness rather than dominating interactions. Analysis of sentiment flow across comment threads showed that early replies were more commonly neutral or positive, often providing reassurance or factual responses, while deeper thread levels exhibited greater emotional variability. Extended exchanges were more likely to include detailed accounts of uncertainty, disagreement, or ongoing challenges, indicating a transition from initial information-seeking to expression of emotional burden. Topics centered on symptom progression or diagnostic uncertainty showed the most negative sentiment, whereas coping-oriented discussions exhibited more positive polarity, reflecting adaptation and problem-focused framing rather than absence of burden.

Highly active contributors primarily posted neutral, information-oriented comments, with relatively few instances of explicit help-seeking or support-offering. While prior work suggests that highly active users may function as informed peers (Simoni et al. 2020), the most active contributors in this dataset mainly facilitated information exchange rather than sustained emotional support. Similar dynamics have been reported in other geographically dispersed rare-disease communities, where informational needs outweigh emotional reciprocity (Ashtari and Taylor 2023, Miller et al. 2021). These patterns highlight the potential value of hybrid support models that link online forums with patient organizations or clinical services.

Temporal analysis showed that engagement intensified around a limited number of posts rather than through increased posting frequency. Peaks in comment activity reflected heightened interest linked to external events or emerging topics, with mid-2024 activity coinciding with discussions of dermatomyositis, COVID-19, and methotrexate. Neutral, enquiry-driven posts predominated during this period. Although isolated case reports have suggested associations between COVID-19–related inflammation and dermatomyositis (Shimizu et al. 2022) or between mRNA vaccination and polymyositis onset in susceptible individuals (Yılmaz 2024), such reports cannot establish causality. Observed discussion spikes are therefore more likely to reflect increased awareness or concern rather than direct changes in disease incidence.

While the subreddit functioned as a largely constructive and informative environment, potential risks associated with unmoderated peer-generated health content remain. Although misinformation was not evaluated in this dataset, the predominance of help-seeking over support-offering posts indicates that medical advice is frequently requested without guaranteed resolution. Prior research has shown that niche health communities may be vulnerable to unverified claims influencing patient decision-making (Shah 2024). Although this study did not evaluate factual accuracy, the analytical framework provides a means of identifying points of uncertainty, dissatisfaction, or rapid discourse shifts that could inform moderation or targeted resource provision.

Participation in community-driven health spaces has been associated with improved mental health outcomes, including reduced isolation and enhanced sense of belonging (Wairimu 2024). Similar patterns were evident within the myositis subreddit, where shared experiences fostered peer connection. From a clinical and organizational perspective, patterns observed in online discussions may help identify areas of unmet need relating to diagnosis, treatment decision-making, or disease progression. Patient organizations and healthcare providers could use such insights to develop targeted educational resources, moderated engagement opportunities, or accessible communication tools to better support individuals living with myositis.

Several limitations should be acknowledged. The analysis was confined to a single platform and English-language posts, potentially limiting representativeness, particularly given that Reddit users tend to be younger and more likely to have higher formal education than the general population (Pew Research Center 2024). Sentiment classification relied entirely on automated models without human annotation, precluding direct comparison with human judgement or calculation of inter-annotator agreement metrics such as Cohen’s κ. This approach was selected to preserve scalability and reproducibility. Although established sentiment models were used, emerging large language models may offer improved sensitivity to contextual nuance in future work.

Despite a modest number of contributors relative to large platform-wide studies, the corpus comprised over 18 000 conversational elements, making automated NLP appropriate. Additional constraints include the absence of demographic identifiers and the episodic nature of community engagement, which complicate subgroup and longitudinal interpretation. While this analysis focused on textual content, future inclusion of image- or video-based communication may further enrich understanding of patient expression.

Beyond myositis, the methods applied here offer a transferable framework for examining online discussions across other rare and long-term medical conditions, enabling comparative insight into shared challenges, information-seeking behavior, and emotional dynamics within digital health communities.

## Conclusion

This study provides detailed insight into the structure and behavior of the myositis subreddit, demonstrating its role as a community where individuals exchange experiences, seek guidance, and share treatment-related information. Communication was predominantly neutral in tone, with negative sentiment emerging primarily in discussions focused on symptom progression, uncertainty, or difficulties accessing care. Periods of increased engagement coincided with external events, illustrating how real-world developments shape online discourse.

The methodological approach demonstrates the feasibility of applying automated NLP to rare-disease discussions and provides a foundation for future studies conducted over extended timeframes or across additional platforms. The patterns identified offer practical value for healthcare organizations and advocacy groups by highlighting areas of uncertainty, how treatment experiences are framed, and topics that may benefit from targeted informational support.

Although this study did not assess the factual accuracy of shared content, the analytical framework could be extended to identify discussion points where misinformation is more likely to emerge, supporting early moderation or targeted resource provision. Collectively, these findings underscore the value of analysing online patient narratives to inform digital support strategies, strengthen patient communication, and guide development of more responsive and accessible resources for rare-disease communities.

## Supplementary Material

SUPPLEMENTARY_MATERIAL_oqag007

## Data Availability

The datasets used in the analysis are presented in this paper. Individual data are not available owing to Birmingham City University’s policies on data privacy and confidentiality.
