# Supplementary material for: Reddit and rare diseases: what myositis communities tell us about support and struggle
Source: Oxf Open Digit Health. 2026 Apr 11;4:oqag007. doi: 10.1093/oodh/oqag007 (PMC13148158; doi:10.1093/oodh/oqag007)
Supplement: SUPPLEMENTARY_MATERIAL_oqag007 [file supplementary_material_oqag007.docx]

**SUPPLEMENTARY METHODS**

**Supplementary Methods S1. Sentiment Flow Construction and Comment-Depth Analysis**

This section describes the sentiment-flow analysis used to examine how emotional tone varies across comment threads. Comment depth was used as an ordinal indicator of a comment’s hierarchical position within a discussion thread. For each depth level, depth-specific sentiment metrics were computed, including the number of comments, number of unique cleaned comments, mean signed sentiment polarity, and the proportion of positive and negative comments based on RoBERTa sentiment outputs.

Mean sentiment values were visualised across increasing comment depth using LoESS smoothing (frac = 0.4) to characterise overall trends while reducing local variability. Additional depth-stratified summaries of comment volume and sentiment label distributions were used to contextualise the stability and interpretability of observed depth-related sentiment patterns.

**Supplementary Methods S2. BERTopic Clustering, Hierarchical Topic Reduction, and LDA Alignment**

BERTopic was applied to the same de-duplicated, lemmatised post–comment corpus used for LDA to enable direct comparison between topic modelling approaches. Documents were constructed from retained lemmas after stopword removal, domain-specific filtering, and part-of-speech constraints. Documents containing fewer than three tokens were excluded from BERTopic analysis.

Sentence embeddings were generated using the SentenceTransformer all-mpnet-base-v2 model. Base topics were identified using HDBSCAN, configured to favour fewer, larger clusters (min_cluster_size = 150, min_samples = 15, cluster_selection_method = "eom"). Documents not assigned to any cluster were labelled as outliers (topic −1) and retained for downstream analyses.

To facilitate comparison with the six-topic LDA solution, BERTopic base topics were merged into six super-topics. Each base topic was represented by an embedding derived from its top ten keywords, and agglomerative hierarchical clustering with cosine distance and average linkage was applied to group base topics into six higher-level clusters.

Each document inherited the super-topic of its base topic. Super-topics were aligned with LDA by assigning each super-topic the dominant LDA topic label among its member documents. This alignment enabled direct quantitative comparison between BERTopic and LDA while preserving the unsupervised nature of both models.

**Supplementary Methods S3. Variance Explanation and Regression-Based Sentiment Modelling**

Ordinary least squares (OLS) regression models were used to assess the proportion of variance in RoBERTa-S sentiment scores explained by topic structure. Models were fitted using raw sentiment scores as the dependent variable and categorical indicators of LDA topic membership and BERTopic super-topic membership as predictors.

For exploratory visualisation, sentiment scores were additionally standardised (z-scored across all documents) and aggregated within LDA × BERTopic topic intersections to support comparative heatmap visualisation; these standardised values were not used in inferential analyses

**Supplementary Methods S4. Programmatic Selection of Illustrative Comments**

Illustrative comments were selected programmatically from the processed dataset using sentiment outputs from the RoBERTa-S classifier. No manual selection, qualitative coding, or interpretive filtering was performed.

Comments were eligible for selection if they had (i) a valid comment_depth value and (ii) non-missing RoBERTa sentiment outputs used to compute signed sentiment (positive scores for POSITIVE labels and negative scores for NEGATIVE labels). Comments were then stratified into depth regions consistent with the quantitative analysis (0–2, 3–10, 11+). Within each region, comments were ranked by signed sentiment and the most positive and most negative examples (top N per region) were extracted for reporting and verification. Extracted excerpts were used solely to illustrate model-predicted sentiment extremes in relation to quantitative depth patterns and were not subjected to qualitative thematic analysis.

**Supplementary Methods S5. Keyword Filtering and Part-of-Speech Constraints**

To support reproducibility of the keyword extraction and topic-modelling pipeline, this section documents the complete filtering strategy applied to the lemmatised corpus prior to frequency analysis and topic modelling.

S5.1 Generic Verb Stopwords (Removed)

*get, go, say, know, see, make, take, think, come, try, tell, want, find, need, look, give, seem, ask, leave, keep, use, happen, show, understand, become, put, talk, stop, hear, mean, let, send, thank, read, bad, normal, good*

S5.2 Custom Domain-Neutral Terms (Removed)

*thing, issue, lot, day, year, time, feel, people, life, help, point, friend, good, bad, much, last, high, low, experience, cause, month, week, many, able, severe, positive, problem, well, way, similar, top, ill, sick, eat, right, patient, weight, normal, mom, parent, kid, include, loss, long, example, due, lose*

S5.3 Retained Clinical Action Verbs

*diagnose, test, lose, walk, eat, experience, include, work, move, cause, struggle*

S5.4 Part-of-Speech (POS) Filtering Strategy

POS filtering was applied using the Natural Language Toolkit (NLTK). Only nouns, adjectives, and the approved clinical verbs listed above were retained to emphasise symptoms, diagnostic elements, body regions, disease descriptors, and treatment-related terminology.

S5.5 Reproducibility Note

All filtering rules, stopword lists, and POS constraints were applied consistently across posts and comments and across all downstream analyses, including keyword frequency analysis, topic modelling (LDA and BERTopic), and top-contributor lexical profiling.

**SUPPLEMENTARY RESULTS**

Supplementary Table S1 shows comparative performance metrics for all sentiment models evaluated in the study, calculated at the unique-text level for posts and comments. Reported metrics include mean signed polarity for posts and comments, average Pearson and Spearman correlations with other models, and mean absolute percentage error (MAPE), reflecting relative disagreement between model outputs rather than accuracy against a ground-truth label. Results are shown for lexicon-based models, transformer-based models, and the lexicon ensemble. These metrics support assessment of internal model consistency, cross-model agreement, and polarity behaviour, and inform the selection of the lexicon ensemble, RoBERTa-S, and RoBERTa-C for downstream analyses.

**Table S1.** Summary of Sentiment Model Performance Across Unique Texts

| **Model** | **Type** | **Mean Polarity (Posts)** | **Mean Polarity (Comments)** | **Pearson Avg** | **Spearman Avg** | **MAPE Avg** | **Notes** |
| --- | --- | --- | --- | --- | --- | --- | --- |
| VADER | Traditional | -0.310 | 0.155 | 0.383 | 0.386 | 0.601 | Negative on posts; improves when normalized. |
| TextBlob | Traditional | 0.019 | 0.066 | 0.370 | 0.377 | 0.633 | Slight positive bias; neutral biased. |
| Afinn | Traditional | -0.058 | 0.057 | 0.354 | 0.384 | 0.628 | Mid-range performance; weaker than VADER/TextBlob mix. |
| Lexicon Ensemble (VADER + TextBlob) | Traditional (combined) | -0.000 | -0.000 | 0.446 | 0.451 | 0.575 | Best classical model stability; strongest agreement among lexicons. |
| RoBERTa (Siebert) | Transformer | -0.429 | -0.102 | 0.457 | 0.494 | 0.656 | Good polarity discrimination; generally more negative. |
| Roberta (Cardiff) | Transformer | -0.477 | -0.201 | 0.490 | 0.513 | 0.548 | Best transformer agreement with other models; stable bias. |
| BioclinBERT | Transformer | 0.539 | 0.539 | 0.198 | 0.210 | 0.723 | Poor polarity range; heavily compressed middle output. |
| DistilBERT Emotion | Transformer | 0.809 | 0.820 | 0.032 | 0.019 | 0.908 | Captures emotions well but polarity mapping is unreliable. |

**Supplementary Results S2. Topic-Level Sentiment Illustrated with Indicative Comments**

To contextualise quantitative topic–sentiment patterns, indicative comments were extracted programmatically from the processed dataset using sentiment scores generated by the RoBERTa-S classifier and topic assignments derived from the six-topic LDA model. Comment selection was fully automated and based solely on model outputs; no manual curation, qualitative coding, or interpretive analysis was performed.

For each LDA topic, three types of exemplar comments were identified:

(i) the comment with the most negative signed sentiment polarity,

(ii) the comment with the most positive signed sentiment polarity, and

(iii) a representative comment with sentiment closest to the mean polarity for that topic.

Signed polarity values reflect RoBERTa-S probability scores, with positive values corresponding to POSITIVE labels and negative values corresponding to NEGATIVE labels.

**Autoimmune Diagnosis, Limb Weakness & Systemic Symptoms**

Within this topic, the most negative comments (polarity ≈ −0.100) included explicit negative expressions relating to healthcare experiences, such as the phrase “*I’m officially p****d*” which appeared repeatedly across duplicated entries. The most positive comments (polarity ≈ 0.999) described clinical encounters involving specialist assessment and diagnostic consideration. Representative comments, with polarity values close to the topic mean (mean ≈ −0.576), described ongoing muscle pain, weakness, and fatigue, often framed as gradual functional decline.

**Localised Organ Symptoms (Chest, Skin, Vision)**

This topic exhibited the most negative mean sentiment overall. The most negative comments (polarity ≈ −0.100) consisted of repeated entries describing anxiety associated with visual symptoms and upcoming clinical appointments. The most positive comments (polarity ≈ 0.999) still referenced substantial symptom burden (e.g. nerve pain or arthritis) but were classified as positive by RoBERTa-S, reflecting linguistic framing rather than symptom severity. Representative comments (mean ≈ −0.647) described recurring organ-specific symptom patterns, including congestion and breathing difficulties.

**Physical Weakness, Fatigue, Sleep & Daily Functioning**

Highly negative comments in this topic (polarity ≈ −0.100) included repeated statements describing dissatisfaction with workplace or healthcare interactions. The most positive comments (polarity ≈ 0.998–0.999) focused on practical adaptations or treatment changes, such as medication adjustments or the use of assistive strategies. Representative comments selected near the topic mean (mean ≈ −0.638) described persistent leg pain, fatigue, and declining physical capacity, consistent with a sentiment distribution skewed toward negative values.

**Healthcare Navigation, Appointments & Treatment Pathways**

The most negative comments (polarity ≈ −0.100) described adverse experiences related to medical appointments and care pathways, often repeated across duplicates. In contrast, the most positive comments (polarity ≈ 0.999) were support-oriented messages addressed to other community members. Representative comments (mean ≈ −0.576) reflected ambivalence around prolonged diagnostic processes and decisions about sharing illness experiences publicly.

**Functional Disability, Life Impact & Social Roles**

In this topic, the most negative comments (polarity ≈ −0.100) included repeated references to limited support from family members and dissatisfaction with clinical responses to reported symptoms. The most positive comments (polarity ≈ 0.999) highlighted social connection, psychological support, or parenting experiences in the context of chronic illness. Representative comments (polarity range ≈ −0.650 to −0.711) described ongoing functional limitations alongside partial symptom management.

**Anxiety, Diet, Somatic Concerns & Coping Behaviours**

This was the only topic with a positive mean sentiment. The most negative comments (polarity ≈ −0.100) referenced limited perceived support from close contacts. The most positive comments (polarity ≈ 0.999) described adaptive behaviours and lifestyle changes. Representative comments near the topic mean (mean ≈ 0.58) described clusters of somatic symptoms (e.g. dizziness or severe episodes) expressed in a comparatively neutral or coping-oriented linguistic style.

**Notes on Interpretation**

The comments presented are intended solely to illustrate model-derived sentiment patterns within topics. They reflect RoBERTa-S sentiment classification and topic membership rather than inferred emotional states or narrative intent. These excerpts were not used for qualitative analysis and do not constitute thematic interpretation beyond their role as illustrative examples of quantitative results.
